# Large scale generation of micro-droplet array by vapor condensation on mesh screen piece

**DOI:** 10.1038/srep39932

**Published:** 2017-01-05

**Authors:** Jian Xie, Jinliang Xu, Xiaotian He, Qi Liu

**Affiliations:** 1 The Beijing Key Laboratory of Multiphase Flow and Heat Transfer for Low Grade Energy Utilization, North China Electric Power University, Beijing, 102206, P.R. China

## Abstract

We developed a novel micro-droplet array system, which is based on the distinct three dimensional mesh screen structure and sintering and oxidation induced thermal-fluid performance. Mesh screen was sintered on a copper substrate by bonding the two components. Non-uniform residue stress is generated along weft wires, with larger stress on weft wire top location than elsewhere. Oxidation of the sintered package forms micro pits with few nanograsses on weft wire top location, due to the stress corrosion mechanism. Nanograsses grow elsewhere to show hydrophobic behavior. Thus, surface-energy-gradient weft wires are formed. Cooling the structure in a wet air environment nucleates water droplets on weft wire top location, which is more “hydrophilic” than elsewhere. Droplet size is well controlled by substrate temperature, air humidity and cooling time. Because warp wires do not contact copper substrate and there is a larger conductive thermal resistance between warp wire and weft wire, warp wires contribute less to condensation but function as supporting structure. The surface energy analysis of drops along weft wires explains why droplet array can be generated on the mesh screen piece. Because the commercial material is used, the droplet system is cost effective and can be used for large scale utilization.

Micro/nano droplet array has many engineering applications. For instance, Zhu *et al*. reported a nanoliter droplet array-based method to quantify gene expression in individual cells^1^,^2^. By sequentially printing nanoliter-scale droplets on a microfluidic robot, all liquid-handling operations including cell encapsulation, lysis, reverse transcription, and quantitative PCR with real-time fluorescence detection, can be automatically achieved. Droplet-based microfluidic system was used to obtain lens effect by using a thin liquid layer on a polar electric crystal like LiNbO_3_^3^. An array of liquid micro-lenses was generated by electrowetting effect in pyroelectric periodically poled crystals. A capacitive-type touch sensor used liquid metal (LM) droplet array^4^. The microsystem has three components including a thin oxide film bottom layer patterned with electrodes, a pyramid-shaped polydimethylsiloxane (PDMS) chamber containing LM droplets, and a top membrane with an electrode. The overlap area between LM droplet and a flat-bottom electrode can be changed to modulate the sensor capacitance.

Self-organized droplet array can be found in bio-engineering for sample handling, test and analysis, optical engineering for image construction, and electronic industries. Modulation of drop growth and movement has made great progress in recent years. He *et al*.^5^ reported a high-efficient removal method for condensed water drops with hierarchically structured porous aluminum surfaces. Zhang *et al*.^6^ shows that the drop contact angles can be determined by the local structure of heterogeneous surfaces. Lv *et al*.^7^ demonstrated the bio-inspired strategies for anti-icing purpose. Zhang *et al*.^8^ investigated enhanced jumping of condensed water droplets by anti-icing surfaces. Liu *et al*.^9^ controlled self-propelled leaping of droplets along a prescribed direction on a micro-anisotropic superhydrophobic surface. We note that droplets can be modulated in a closed system. Huang *et al*.^10^ demonstrated the on-site formation of emulsions by controlled air plugs. Alternatively, droplets can also be generated and modulated in an open environment. Hou *et al*.^11^ showed the spatial formation of droplet on a hybrid hydrophic/superhydrophobic surface.

The generation and modulation of droplets are based on micro/nano system. The substrate material for these systems is silicon^12^, glass^13^ and PDMS^14^. These systems have a common nature of compact size. The overall chip size is in ~mm-cm scale. The batch-fabrication of microsystems is helpful to reduce the fabrication cost. Generation of micro/nano droplet array on a large surface is a challenge issue. How can we produce micro/nano droplet array on a ~m^2^ scale metallic surface? The techniques of droplet pattern formation for microsystems are difficult to be introduced for large scale utilization. The cost will be significantly increased when one shifts the microsystem based droplet generation technique to large scale utilization. The mechanical engineering does need such requirement. If one machines hydrophilic dots on a large scale super-hydrophobic copper surface, condensation heat transfer will be improved due to the delayed liquid flooding on the surface^15^. Very recently, Yoo *et al*. noted that periodic water droplets on a piece of metallic surface can be used to absorb electromagnetic wave, which is attractive for military fighter, tank and warship to avoid radar detection^16^.

Here, we reported a novel micro-droplet pattern mechanism in large scale on metallic surface by dropwise condensation. The device works based on the distinct three dimensional (3D) mesh screen structure, which was sintered on a copper substrate. Only bottom part of weft wires are soldered on the copper substrate, inducing non-uniform residue stress along curved weft wires. The outer and inner sides of weft wires exhibit pulling and compressive stresses, respectively. The stress is larger at the weft wire top location than elsewhere. When the sintered structure is oxidized, micron sized pits exist and there are no much nano-grasses on the weft wire top area due to the over-oxidation there. Nano-grasses grow on other locations of weft wires to show hydrophobic behavior. Thus, novel surface-energy-gradient weft wires are formed periodically. Cooling the copper substrate and mesh screen package in a wet air environment nucleated water droplets on the weft wire top location, which is more “hydrophilic” than other locations. Changing the cooling water temperature, cooling time or air humility modulate droplet size. Warp wires had higher temperatures than weft wires thus droplet nucleation seldom happens on warp wires. Because the material (copper and mesh screen) is commercialized and the sintering technique is mature, the device is cheap and suitable for large scale generation of droplet array.

## Non-uniform stresses along weft wires

**Figure 1:**
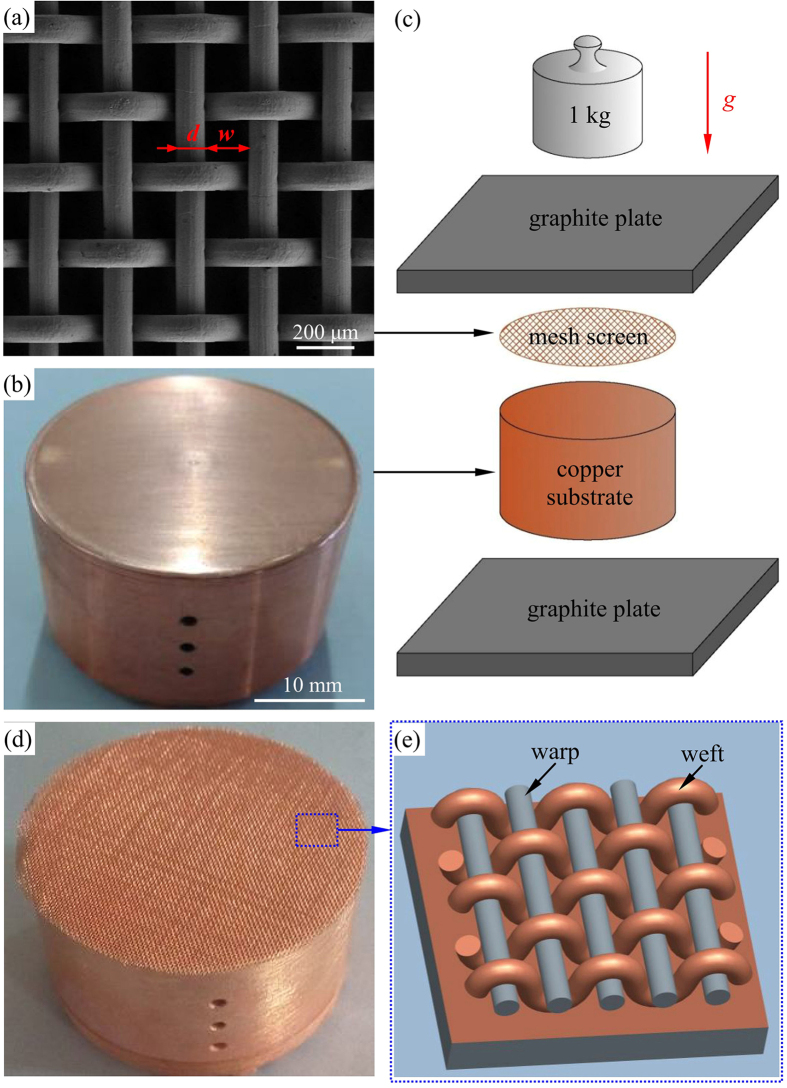
The mesh screen sintered on copper substrate. (**a**) Mesh screen, (**b**) copper substrate, (**c**) bonding the two components by applying external force, (**d**) Mesh screen sintered on the copper substrate, (**e**) enlarged test section structure before oxidation.

Preparation of test section consists of two steps: sintering a mesh screen piece on a copper substrate, and test section surface modification to create micro/nano structure. Figure 1 shows the sintering process before oxidation. The copper mesh screen is widely used in industries such as heat pipes^17^ and gas-liquid separator^18^. The mesh wire diameter is *d* and the rectangular pore width is *w (d* = 101 μm and *w* = 152 μm here, see Fig. 1a). The distinct three dimensional structure consists of straight warp wires and curved weft wires. In other words, the mesh screen had parallel and curved weft wires separated by straight warp wires, periodically. A copper cylinder block with a diameter of 30 mm and a height of 20 mm was carefully machined and polished by fine abrasive paper (see Fig. 1b).

The mesh screen is put on the top of the copper substrate. The two components are bonded together by imposing an external force generated by a graphite mould and a weight of 1 kg (see Fig. 1c). The compression stress induced by the weight is important to keep the close contact between mesh screen and copper substrate. This also ensures the local area soldering (not a point soldering) of weft wires with the copper substrate, maintaining relatively uniform temperatures between weft wires and copper block. Figure 1d shows the test section after sintering. The enlarged 3D structure was shown in Fig. 1e, noting that bottom part of weft wires are soldered with copper substrate. Warp wires are suspended in the air environment, but separate weft wires periodically as the supporting structure.

**Figure 2:**
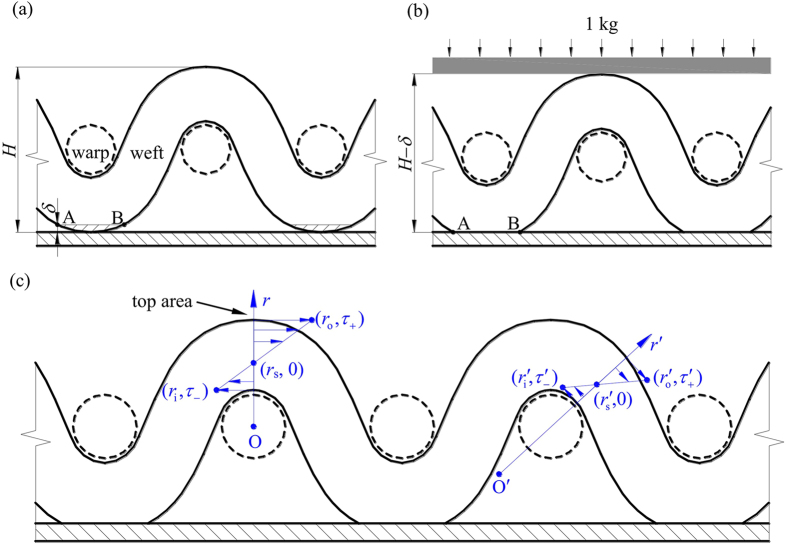
The stress analysis after the sintering process. (**a**) Mesh screen on the copper substrate before applying external force, (**b**) mesh screen weak deformation after applying external force, (**c**) stress analysis at the top area and elsewhere deviating from the top area after the sintering process when the weight was removed.

The sintering process under applied external force caused non-uniform stresses along curved weft wires. Figure 2a shows a mesh screen on a planar surface without external force, behaving point contact between them. When we apply an external force such as 1 kg on the mesh screen piece, the initially suspended AB points (see Fig. 2a) contact the planar surface due to the soft deformation of the material (see Fig. 2b). Correspondingly, the mesh screen height is changed from *H* to *H*–*δ* indicating a *δ* deformation in the height direction. When the external force (weight) was removed from the mesh screen, residue stress occurs. Figure 2c shows a center cross section of weft wire, periodically separated by parallel warp wires, after the sintering process. The weft wire top location was paid attention first. A coordinate system was established as O as the original point and *r* as the radius index. The stress is expressed as^19^


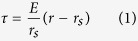


where *E* is the modulus of elasticity, *r*_*s*_ is the radius at the zero stress. The positive and negative *τ* indicate the pulling stress and compression stress, respectively. The outer side (*r* > *r*_*s*_) and inner side (*r* < *r*_*s*_) behave pulling and compression stresses, respectively. However, when the location deviates from the top location, the original point shifts to O′ and the radius is marked by *r*′. The stress is


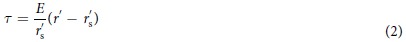


Because the curvature radius *r*_*s*_ at the top location is smaller than that at other locations, the surface at the top location has larger pulling stress than elsewhere. Thus, a non-uniform stress along weft wires is formed. The maximum pulling stress occurs on the weft wire top location. Due to the distinct structure of the mesh screen, the distance between two maximum pulling stress points is 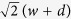.

Seeing Figs 1e and 2, we observed the external force applied on weft wires but not on warp wires. The weft and warp wires have identical diameter, but their spatial arrangements are totally different. Weft wires are curved in the membrane height direction but warp wires are suspended parallel to the planar copper surface. The external force directly applied on weft wire top locations causes micro-deformation, yielding residual stress along weft wires after the sintering process. However, when the external force is applied on the mesh screen piece, the center of mass along different axial locations of warp wire will be pulled down a same height, which should be very small. Thus, there is no deformation at all along warp wires to result in any force and stress on warp wires. The chemical treatment of the sintering package under the residual stress condition is the reason to form the surface-energy-gradient weft wires.

## Surface-energy-gradient weft wires

**Figure 3:**
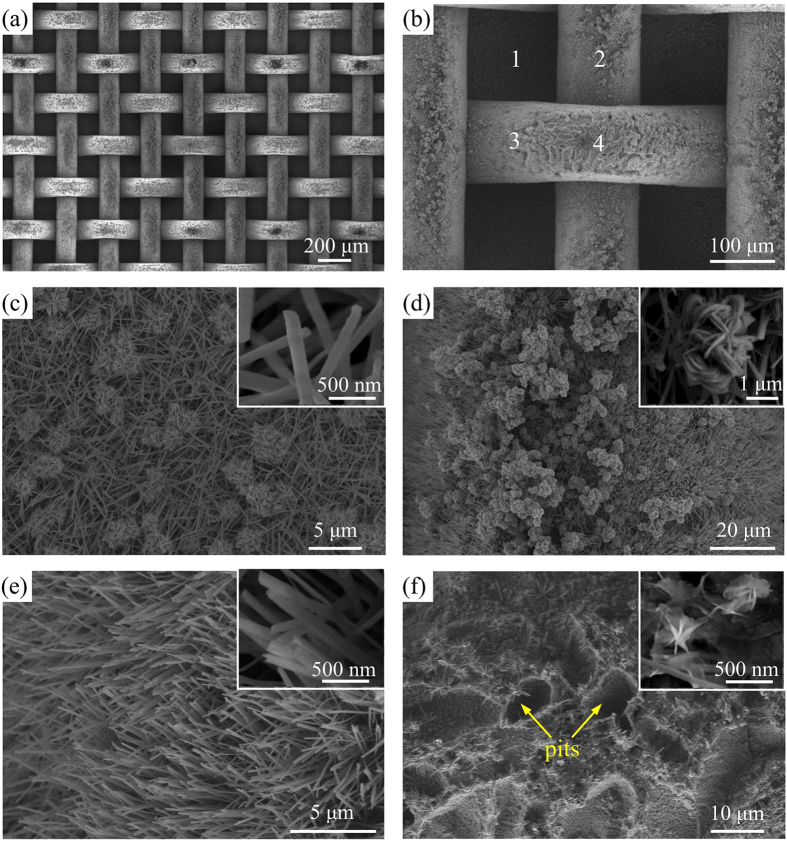
SEM images after the chemical treatment of the mesh screen and copper substrate. (**a**) Mesh screen, (**b**) four regions, (**c**) region 1 on copper surface area, (**d**) region 2 on warp wire surface, (**e**) region 3 on weft wire surface area deviating from the top location, and (**f**) region 4 on weft wire top location, densely populated nanorods are on region 3 and micro-pits with few nanograsses are on region 4, respectively.

The sintered sample was oxidized and modified referring to Chen *et al*.^20^ and Feng *et al*.^21^ (See details in the section of methods). A gradually enlarged SEM (scanning electron microscope) images are shown from Fig. 3a to f for the finally obtained sample. Figure 3b illustrates a typical unit of the imaged area, in which regions 1, 2, 3 and 4 referred to copper substrate area, warp wire surface area, weft wire surface deviating from the top location, and weft wire top location, respectively. The copper substrate and warp wire surface behaved CuO flower-like microstructure and nanorod arrays (see Fig. 3c,d). The enlarged SEM images show the micro-flower has a diameter of 2–5 μm while the nanorod has a diameter of about 100 nm.

It is interesting to note different micro/nano structure on different locations of weft wire. Region 3 (see Fig. 3e) is located on the weft wire surface deviating from the top location. Densely nano-grasses are seen to behave the super-hydrophobic characteristic. Nano-grasses are seen to be inclined due to the curved weft surface. However, Fig. 3f shows that, on the weft wire top location (region 4 in Fig. 3b), the stress induced micro/nano structure can be seen. During the chemical treatment of the mesh screen sample, the weft wire top location is over oxidized to form pits^22^, having ~10 μm size. The micro pits structure with less nanograsses cause more “hydrophilic” than its neighboring area. The 3D ordered mesh screen structure forms the stress induced pits array.

The mesh screen sintering on copper substrate under external force forms non-uniform residual stresses along weft wires after sintering. Maximum stress exists at the weft wire top location. Oxidation of the mesh screen with non-uniform stress generates surface-energy-gradient weft wires. Droplet nucleation will take place on micro-pits array on weft wire top location due to “hydrophilic” behavior related to the neighboring hydrophobic area. This is the main mechanism to form micro droplet pattern on mesh screen.

## Results and Discussion

### Self-organized micro/droplet array

**Figure 4:**
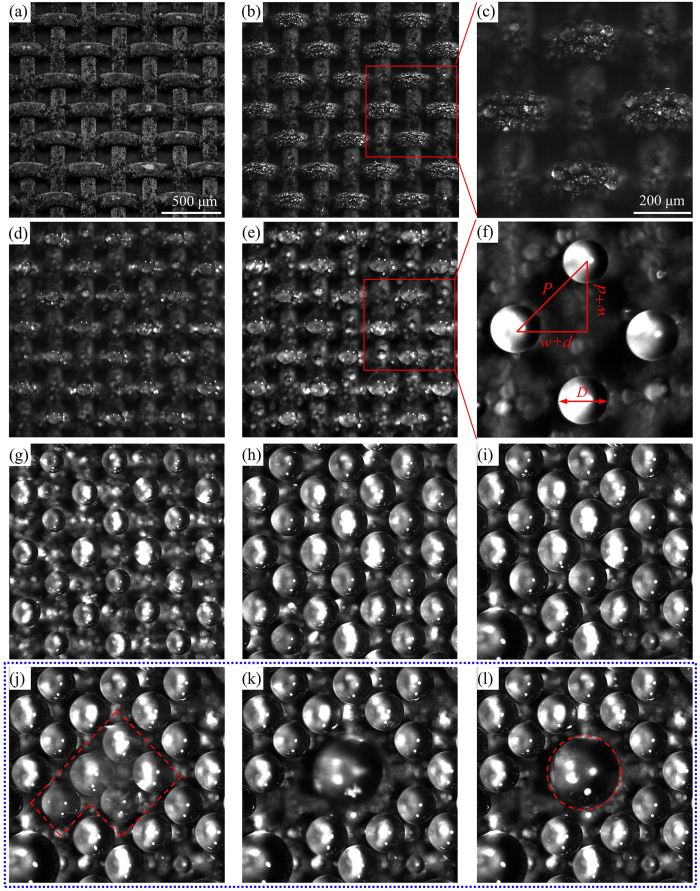
Drop growth process with *RH* = 40%, *T*_e_ = 26.0 °C, and *T*_sub_ = 6.0 °C. (**a**,**b**,**d**,**e**,**g**,**h**,**i**) Represent drop growth process with consecutive time difference of 15 minutes, (**c**) for enlarged (**b**,**f**) for enlarged (**e**,**j**,**k**,**l**) represent the coalescence when the drops attain the coalescence criterion with sequence time of 10 ms.

The condensation experiment was performed. Figure 4 shows the dynamic process of the self-organized condensation droplet array with environment temperature of *T*_e_ = 26 °C, air humidity of *RH* = 40.0% and cooling substrate temperature of *T*_sub_ = 6.0 °C. The subfigures of a, b, d, e, g, h and i illustrate the dynamic images with consecutive time difference of 15 minutes between neighboring subfigures. The subfigures of c and f are locally enlarged images for b and e, respectively. Figure 4j–l shows the drop coalescence process with consecutive time of 10 ms.

The time *t* = 0 is defined as the start of the cooling process (see Fig. 4a). At *t* = 15 min, tiny drops appeared on weft wire top locations but drops are seldom observed on warp wires (see Fig. 4b–c). Oxidization of weft wires with non-uniform stress forms “hydrophilic” pits area, which became water drop nucleation sites during condensation. Coalescence of tiny drops on weft wire top location generate self-organized droplet array pattern (see Fig. 4d,e,g,h and i). The droplet diameter is *D*, which is being increased by continuous cooling on the copper substrate. Four droplets form a unit of droplet array (see Fig. 4f). 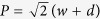 is the distance between two centers of neighboring droplets and does not change with time. The maximum droplet diameter is 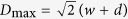, beyond which drop coalescence occurs (see Fig. 4j–l). The similar dynamic process of the self-organized condensation droplet array for air humidity of *RH* = 60.0% and substrate temperature of *T*_sub_ = 1.0 °C can be found in Supplementary Movie 1.

**Figure 5:**
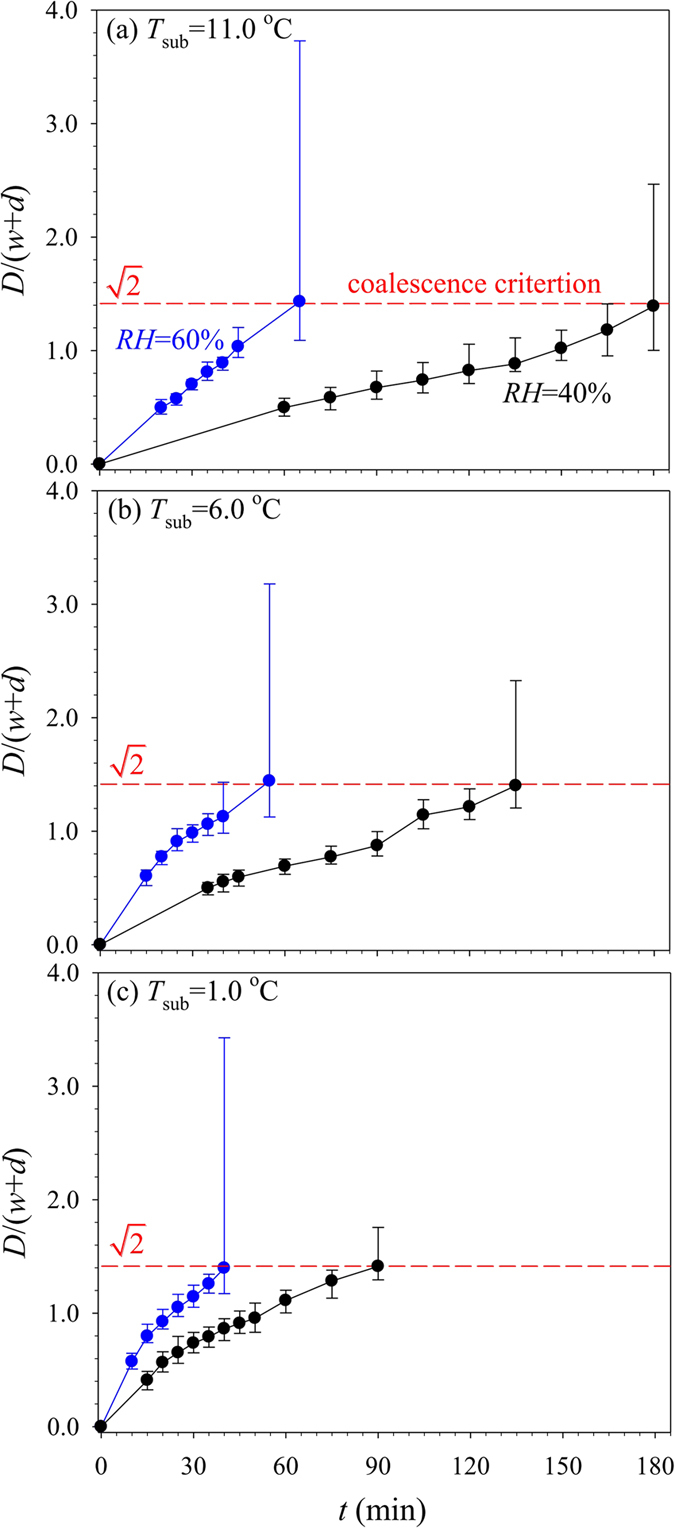
Drop sizes dependent on *RH, T*_sub_ and cooling time *t.*

Figure 5 shows non-dimensional drop diameters, 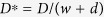, versus cooling time *t*, cooling temperature *T*_sub_ and air humility *RH.* It is seen that 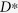 is increased by *t*. The transition criterion is 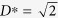 or 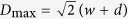. Increasing air humidity *RH*, and/or decreasing cooling temperature *T*_sub_, accelerate the drop growth. Quasi-uniform drop sizes are obtained. The difference between maximum and minimum diameters divided by the average value is termed as the drop size deviation, which is less than 8% for all of the tests.

### Heat transfer analysis

The condensation theory^23^ gave the minimum energy barrier of nucleation as


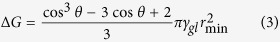


where *θ* is the contact angle, *γ*_gl_ is the surface tension between gas and liquid, *r*_min_ is the minimum nucleation size^24^,^25^:


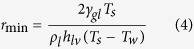


where *T*_s_ is the saturation temperature of vapor, *h*_lv_ is the latent heat of evaporation and *T*_w_ is the wall temperature. Drop nucleation site is preferred to occur on hydrophilic surface, caused by decreased Δ*G* with decrease of *θ*. The weft wire top location satisfies the necessary condition because it is more “hydrophilic” than elsewhere. *T*_w_ should be sufficiently low to minimize the energy barrier. The heat transfer analysis is to (1) verify if the weft wire top location has similar temperature as that of the copper substrate; (2) estimate heat transfer rate via weft wire; (3) evaluate heat transfer rate via warp wire to see if it is much smaller than that on weft wire. If the three objectives are reached, we fully understand why the nucleation sites are preferred to happen on weft wire top locations, and warp wires contribute less to condensation.

**Figure 6:**
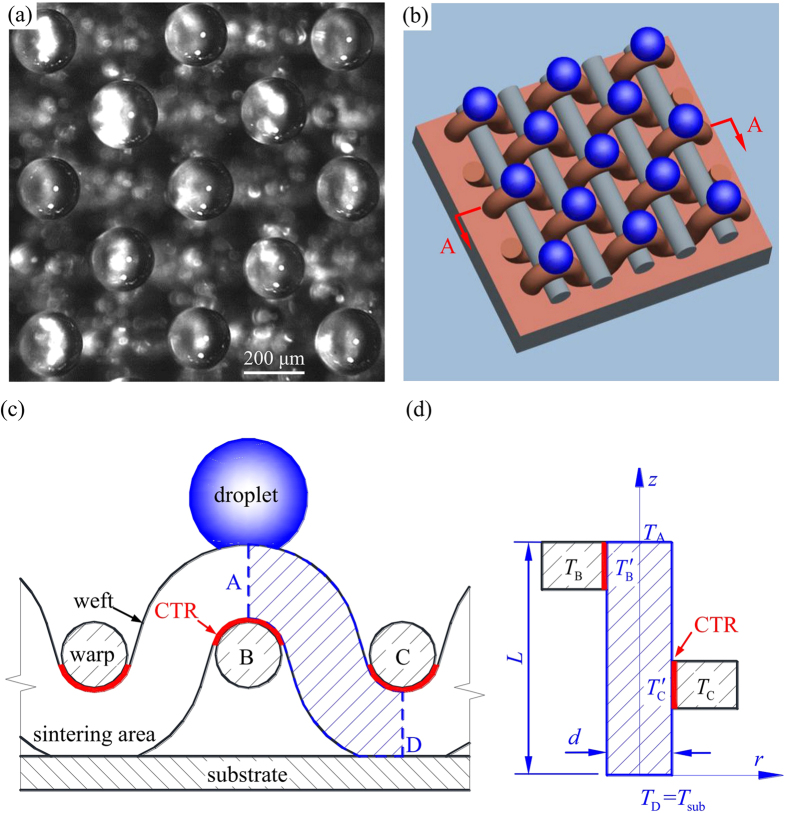
Heat transfer model explaining why drops are generated on weft wires and warp wires contribute less to condensation. (**a**) Photo of micro drop array, (**b**) 3D view of micro drop array, (**c**) physical model of mesh screen sintered on copper substrate, (**d**) thermal resistance analysis.

Figure 6a,b shows typical micro-droplet array on mesh screen, which is caused by the 3D mesh screen structure and distinct thermal/fluid performance. A unit of mesh screen was selected to have a project area of (*w* + *d)*^*2*^, where *w* and *d* are pore width and mesh wire diameter, respectively. Both weft wire and warp wire are exposed in a wet air environment with a temperature of *T*_e_. Based on the correlation^26^, nature convective heat transfer coefficient is estimated to be *h*_air_ = 22 W/m^2^K without condensation. Alternatively, according to Ma *et al*.^27^, the condensation heat transfer coefficient is *h*_air,c_ ~ 1000 W/m^2^K. The Biot number characterized as *Bi* = *h*_air,c_*d*/λ_cu_ = 7.6 × 10^−4^ is much smaller than 1, where λ_cu_ is the copper thermal conductivity. Thus, temperatures are very uniform in the mesh wire radius direction. We only consider temperature distribution along axial mesh wires. Figure 6c shows physical model for heat transfer analysis. The shaded blue area represents the curved weft wire but black areas represent warp wires B and C. The curved weft wire was stretched into a straight cylinder fin with its diameter of *d* = 101 μm and height of *L* = 402 μm. The cylinder fin was sintered on the copper substrate surface with its bottom temperature of *T*_D_ = *T*_sub_. The cylinder surface is exposed in wet air environment with the temperature of *T*_e_. Warp wires did not contact copper substrate and there is a conductive thermal resistance (CTR) between warp wire and weft wire (see Fig. 6d). Momentarily, we do not consider heat transfer between warp and weft wires.

Based on fin heat transfer model^28^, the excess temperature is defined as *ϕ*_*z*_ = *T*_e_ − *T*_*z*_, which is


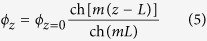


where 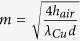. At the fin top, the excess temperature is


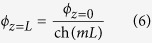


Substituting *h*_air_ = 22 W/m^2^K or *h*_air,c_ = 1000 W/m^2^K into *m* and with *L* = 402 μm, 1/ch(*mL*) equals to 0.9998 and 0.9920, respectively. Both 1/ch(*mL*) approach 1, indicating very small temperature difference between fin top and copper substrate.

Within a project area of (*w* + *d*)^2^, the heat transfer rate via weft wire surface is


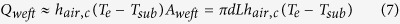


Noting that in Eq. 7, the heat transfer area *A*_weft_ is π*dL*, where *L* is the fin height. Giving *h*_air,c_ = 1000 W/m^2^K and *T*_e_ = 26.0 °C, *Q*_weft_ is 3.16 × 10^−3^ W, 2.53 × 10^−3^ W, and 1.89 × 10^−3^ W for three *T*_sub_ of 1.0 °C, 6.0 °C and 11.0 °C, respectively.

Now we consider heat transfer rate via warp wire surface within a project area of (*w* + *d*)^*2*^. The key factor to determine such heat transfer rate is the conductive thermal resistance (CTR) of air between warp and weft wires (see Fig. 6c). Based on the intercrossed contact between warp and weft wires, the average gas film thickness was given as


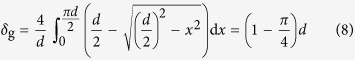


Correspondingly, CTR is 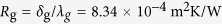, which is three orders larger than the conductive thermal resistance of warp wire 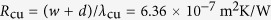. The heat transfer rate via warp wire surface is


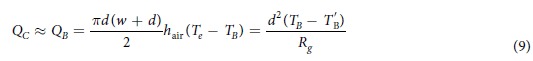


where 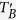 is the temperature on weft wire surface that contacts the warp wire B (see Fig. 6d), 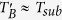. Giving *h*_air_ = 22 W/m^2^K and *T*_e_ = 26.0 °C, *T*_B_ is 2.1 °C, 6.9 °C and 11.6 °C for three *T*_sub_ of 1.0 °C, 6.0 °C and 11.0 °C, respectively. It is seen that warp wire temperatures *T*_B_ are higher than those on weft wires. Thus, drop nucleation sites are preferred to occur on weft wire surface. Based on Eq. 9, *Q*_B_ or *Q*_C_ on warp wire surface is 2.02 × 10^−4^ W, 1.62 × 10^−4^ W, and 1.21 × 10^−4^ W for three *T*_sub_ of 1.0 °C, 6.0 °C and 11.0 °C. *Q*_B_ or *Q*_C_ is one order smaller than *Q*_weft_ on weft wire surface. The analysis supports our observation that fewer droplets are seen on warp wires (see Fig. 4).

### Coalescence of droplets due to surface-energy-gradient weft wires

**Figure 7:**
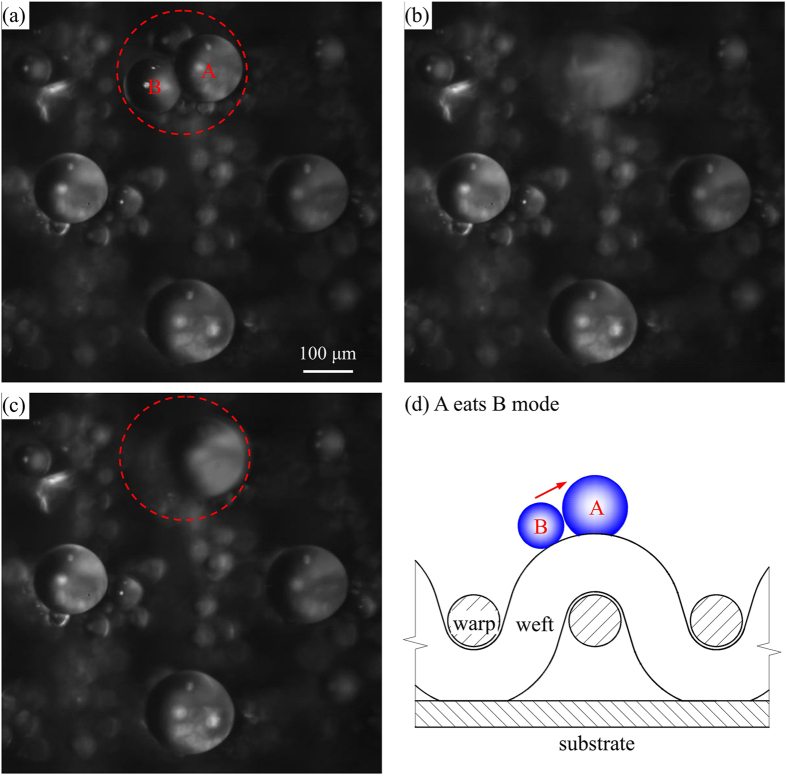
Fast drops coalescence with *RH* = 40%, *T*_e_ = 26.0 °C and *T*_sub_ = 1.0 °C. (**a**,**b** and **c**) are for time difference of 1 ms, (**d**) shows the physical model of two drops coalescence.

We analyzed drop coalescence along curved weft wire surface, which is one of the mechanisms to form self-organized drop array on mesh screen. Figure 7a–c shows two neighboring drops coalescence sequence, noting 1 ms of the time difference between two images. The dynamic process can be found in Supplementary Movie 2. The physical picture is shown in Fig. 7d, in which a “larger” drop A stays at the weft wire top location, but a “smaller” drop B stays at the neighboring location. The coalescence of the two drops is called “A eats B mode”, caused by the surface energy gradient of the weft wire.

A single drop was considered first. Assuming spherical drop, the ratio of a drop surface energy (*G*) related to its gravitational potential energy (*W*), *K*, is


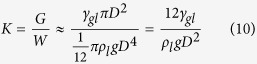


where *γ*_gl_ is the surface tension force between gas and liquid, *ρ*_*l*_ is the liquid density and *g* is the gravity acceleration. Having 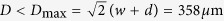, *γ*_gl_ = 72.6 mN/m, *ρ*_*l*_ = 998 kg/m^3^, *g* = 9.8 m/s^2^, *K* is larger than 695, indicating less importance of the gravitational potential energy (*W*). Thus, *W* is not considered. The Gibbs free energy is^29^


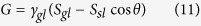


where *θ* is the contact angle, *S*_*gl*_ and *S*_*sl*_ are the gas-liquid contact area and solid-liquid contact area for a drop, which are


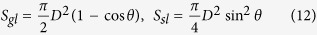


The drop diameter *D*, drop volume *V* and contact angle *θ* have the following relationship


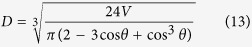


Substituting Eqs 12–13 into Eq. 11, we get


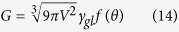


where *f(θ*) is


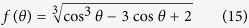


**Figure 8:**
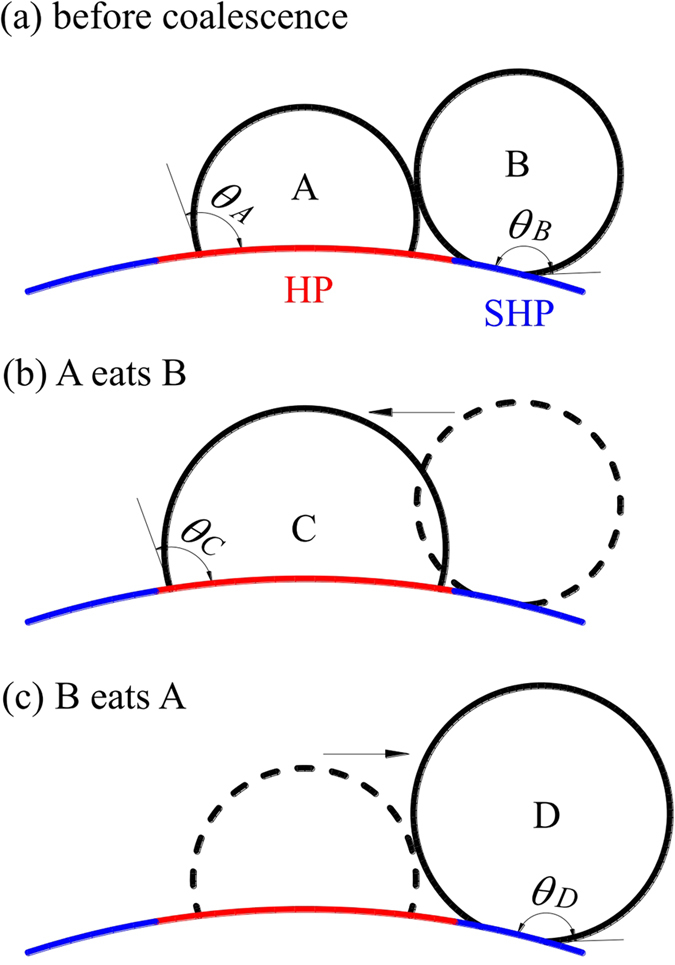
The surface energy analysis for drops coalescence. (**a**) Before coalescence, (**b**) A eats B to form drop C, satisfying the minimum surface energy principle, (**c**) impossible case for “B eats A” to from drop D, not satisfying the minimum surface energy principle, HP and SHP mean hydrophobic and super-hydrophobic, respectively.

Now we consider two drops coalescence. The weft wire top location behaves more “hydrophilic” compared with its neighboring location. Recording the top location as HP (hydrophobic, marked by red color) and its neighboring location as SHP (super-hydrophobic, marked by blue color). Before coalescence, drop A stays at HP with contact angle of *θ*_A_, but drop B stays at SHP with contact angle of *θ*_B_, noting *θ*_A_ < *θ*_B_ (see Fig. 8a). If the drop B has a volume of *ε* times of drop A (*V*_B_ = *εV*_A_). Totally, the two drops have the Gibbs free energy of


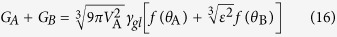


We note the metastable state shown in Fig. 8a Possibly, drop B in SHP area moves towards drop A in HP area to form drop C (see Fig. 8a). Because drop C is within the HP area, the contact angle *θ*_C_ equals to *θ*_A_. At the steady state after coalescence, drop C has the Gibbs free energy of


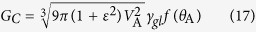


Alternatively, see Fig. 8c, if drop A in HP area moves towards drop B in SHP area to form drop D, the Gibbs free energy of drop D is


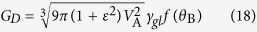


Equation 18 is valid by assuming that the contact angle of drop D in SHP area *θ*_D_ still equals to *θ*_B_. The minimum surface energy principle^30^ determined the pathway choice between Fig.8b and c. In order to compare surface energies expressed in Eqs 16, 17, 18, the derivative of *f(θ*) is


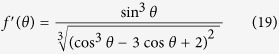


The *f* ′(*θ*) > 0 in the range of *θ* ∈ [0, π] told us that *f(θ*) is increased with increase of *θ*. Comparing Eqs 16, 17, 18 yields *G*_C_ < *G*_D_ and *G*_C_ < *G*_A_ + *G*_B_ to choose the pathway shown in Fig. 8b (called “A eats B” mode). The drop always moves towards the higher energy area when it merges with another drop.

Here, we used the minimum energy principle to explain why the drop automatically moves towards the weft wire top location. The Gibbs surface energy before and after the drop coalescence was paid attention, the drop dynamics such as retraction speed is not considered. When the drop grows larger, the drop shape is influenced by the surface geometry. For the present problem, the three-dimensional weft wire surface makes the problem complicated, which needs further investigation. Liu *et al*.^31^ showed that the asymmetry of the bouncing leads to ~40% reduction in contact time for drop bouncing on curved surface.

Finally, we discuss the drop shape on curved weft wires. The presented Gibbs surface energy analysis helps us to understand why tiny drops move towards the weft wire top location, assuming spherical crown shape for droplets. The analysis is consistent with our experimental observation on drop movement and coalescence. Practically, drop may deviate from the perfect sphere shape on curved mesh wire surface. The drop on curved mesh wire surface is a complicated three-dimensional problem, which involves not only a curvature along the axial mesh wire direction, but also a curvature along the mesh wire circumference direction. The theoretical analysis of the problem is not possible. We recommend the three-dimensional numerical simulation in the future. Zhao^32^ gave the theoretical expression of the drop shape for two-dimensional symmetrical problem, such as drop interaction with planar wall surface and symmetrical convex or concave solid wall surface.

## Conclusions and Perspective

Generation of micro-droplet array on a large metallic surface is a challenging issue. A novel micro-droplet array system was reported using multi-disciplinary knowledge of mechanics, material and thermal/fluid science. Mesh screen was sintered on a copper substrate by applying a specific bonding pressure. Mesh screen is a commercial and cheap material to have micron sized mesh pores for large scale utilization. The structure is periodical, forming the basis to generate droplet array. Non-uniform residual stress exists along curved weft wires after sintering when the external force is removed. There are three mechanisms for the drop nucleation and growth on weft wire top locations: (1) The weft wire top locations are more “hydrophilic” than the neighboring area. (2) The weft wire top locations approach the coldest temperature at the copper substrate. However, the warp wire temperature is higher than the weft wire. Warp wires contribute less to the condensation but function as the supporting structure. (3) Drops coalescence happens along curved weft wire surface due to the surface-energy-gradient.

Mesh screen based droplet generation concept can have many applications. For example, dropwise condensation on solid walls possesses random drop nucleation and growth, in an uncontrolled way. Sintering mesh screen on copper wall not only extends the heat transfer area, but also makes the condensation under a controlled manner to modulate the performance, which is important for precise temperature control applications. Another potential application is for environment engineering. It is difficult to collect ultra-fine dust particles from a large space volume to make the air clean. The study was reported to absorb fine particles by an electriferous single drop^33^. The present work provides a new way to absorb fine dust particles by drop array to protect environment for large scale utilization.

Because modulation of drops has wide applications in mechanical, electrical, optical and biology engineering, droplet dynamics has been received great attention. Chen *et al*.^34^ studied dynamic polygonal spreading of a droplet on a lyophilic pillar-arrayed surface, and shows that their results may expand our knowledge of the liquid dynamics on patterned surfaces and assist surface design in practical applications. Mesh screen is a commercial material to have micro-holes for large scale utilization. Mesh screen will expand its functions with the help of nano-structure modification on the surface. There are affluent phenomena and mechanisms to be explored on the interactions between droplet and mesh screen based micro/nano structure. The perspective research directions are: (1) droplet dynamics on mesh screen based micro/nano structures, including impacting, spreading, bouncing and coalescence, (2) effect of external field such as electric field and magnetic field on the drop dynamics, (3) effect of heat transfer on the drop dynamics, and (4) high resolution experimental and numerical studies on the drop dynamics.

## Methods

### Sintering mesh screen on copper block

A mesh screen piece with mesh wire diameter of 101 μm and rectangular pore width of 152 μm is chosen to sinter on a copper cylinder block with diameter of 30 mm and height of 20 mm. Before the sintering progress, the mesh screen piece was prepared to have identical planar size as that of the copper substrate. The copper cylinder block was carefully machined and polished by fine abrasive paper. Then, both the copper block and mesh screen were cleaned in ultra-sound excited acetone and ethanol solution sequentially, to clean oil contamination. The dried mesh screen is put on the top of the copper substrate. The two components are bonded together by imposing an external force generated by a graphite mould and a weight of 1 kg. All the components are sent into an oven for sintering. The sintering process is described as follows. Initially, the oven is vacuumed to an absolute pressure of 1 kPa. Then the nitrogen gas (*N*_2_) was charged into the oven to have a pressure of 0.5 bar. Under the *N*_2_ protection environment, the sample was sintered at a temperature of 850 °C for one hour. Then, by switching off the oven power, the oven temperature is automatically decreased to 30 °C by 24 hours.

### Oxidization and polymeric modification of the sintered sample

The sintered sample was oxidized by an aqueous solution of 2.5 mol/L sodium hydroxide and 0.1 mol/L ammonium persulphate at room temperature for about 12 minutes, during which the liquid was being mixed by a magnetic stirrer at a 500 r/min speed. The sintered sample was taken out of the solution and fully rinsed by deionized water. Then, the sample was baked in an oven at the temperature of 180 °C for 2 hours. Finally, The oxidized sample is immersed in 0.5 wt% hexane solution of 1 *H*,1 *H*,2 *H*,2*H*-Perfluorodecyltriethoxysilane (FAS17, Alfa Aesar). The reaction time was about 2 hours with the help of a magnetic stirrer. The sample is ready for use after drying in an oven at the temperature of 110 °C for 1 hours.

### Experiment details

The condensation experiment was performed under constant environment temperature *T*_e_ = 26 °C. The air humidity *RH* was also controlled as *RH* = 40% or *RH* = 60.0% by a wet steam humidifier and recorded by a humidity meter. The copper substrate (test section) was put on a cooling block, inside which the chiller water was flowing through. The temperature at the copper substrate surface *T*_sub_ was measured by a thermocouple and recorded by a temperature recorder. The copper substrate surface *T*_sub_ was changed to 1.0 °C, 6.0 °C or 11.0 °C by adjusting the chiller water bath temperature. In such a way, micro droplet array was generated on mesh screen surface under different working conditions. The dynamic growing process of the self-organized condensation droplet array was observed by an optical measurement system, which was included a microscope (SMZ1500, Nikon) bonded with a high speed camera (Motion pro Y4, IDT). The image file was transferred into a computer. The image size covered the pixels of 1016 × 1016. At the maximum optical amplification ratio of 16, the visualization size was 770 μm. Thus, the minimum size resolution was 0.76 μm. During the experiment, the visualization area could be changed manually.

## Additional Information

**How to cite this article**: Xie, J. *et al*. Large scale generation of micro-droplet array by vapor condensation on mesh screen piece. *Sci. Rep.* **7**, 39932; doi: 10.1038/srep39932 (2017).

**Publisher's note:** Springer Nature remains neutral with regard to jurisdictional claims in published maps and institutional affiliations.

## Supplementary Material

Supplementary Information

Supplementary Movie 1

Supplementary Movie 2
