# Supplementary material for: Large scale generation of micro-droplet array by vapor condensation on mesh screen piece
Source: Sci Rep. 2017 Jan 5;7:39932. doi: 10.1038/srep39932 (PMC5215635; doi:10.1038/srep39932)
Supplement: Supplementary Information [file srep39932-s1.pdf]

Supplementary Information

Large scale generation of micro-droplet array by vapor condensation on mesh screen piece

Jian Xie, Jinliang Xu *, Xiaotian He, Qi Liu

The Beijing Key Laboratory of Multiphase Flow and Heat Transfer for Low Grade Energy Utilization, North China Electric Power University, Beijing, 102206, P.R. China

Corresponding author: Dr Jinliang Xu, Tel: 86-10-61772268, email: xjl@ncepu.edu.cn

Supplementary Movies

Supplementary Movie 1:

The dynamic process of the self-organized condensation droplet array in a wet air environment with T_e= 26 ℃, *RH*=60% and T_{sub}= 1 ℃. The field of view is 2.18×2.18 mm.

Supplementary Movie 2:

The dynamic process of the condensation drops coalescence along curved weft wire surface with T_e= 26 ℃, *RH*=40% and T_{sub}= 6 ℃. The video was captured at 1000 fps and played at 2 fps. The field of view is 0.77 mm×0.77 mm.
